# Perinatal outcomes in Malta between 2008 and 2022: a comparison of three 5 yearly epochs

**DOI:** 10.3389/fpubh.2025.1514661

**Published:** 2025-07-02

**Authors:** Nadine Anne De Battista, Miriam Gatt, Minesh Khashu

**Affiliations:** ^1^Department of Child and Adolescent Health, Mater Dei Hospital, Imsida, Malta; ^2^Directorate for Health Information and Research, Malta Congenital Anomalies Registry (MCAR), Guardamangia, Malta; ^3^Neonatal Unit, University Hospitals Dorset NHS Foundation Trust, Poole, United Kingdom

**Keywords:** perinatal, neonatal, outcomes, Malta, public health

## Abstract

**Background:**

Perinatal and neonatal mortality rates are quality indicators of antenatal, neonatal and postnatal care. This study describes perinatal outcomes for Malta over the 15 year period 2008–2022 to inform future practice and healthcare provision.

**Methodology:**

Aggregate anonymous data on perinatal outcomes from 2008 to 2022 were obtained from the National Obstetric Information System (NOIS), Directorate for Health Information and Research (DHIR). Data for each variable was grouped into three 5-year intervals (2008–2012, 2013–2017 and 2018–2022) to investigate trends over time.

**Results:**

Total births increased over time (*p*-value 0.008), mainly driven by singleton pregnancies (*p*-value 0.004), while multiple pregnancies remained stable. Live births increased (*p*-value 0.008), however, there was no statistically significant difference in stillbirth rate. There were no statistically significant changes in post-term, preterm or low birthweight deliveries (*p*-value 0.73). The neonatal mortality rate showed a downward trend from 4.92 per 1,000 live births for the years 2008–2012 to 3.92 per 1,000 births for the 2018–2022 epoch, but this could not be statistically confirmed. Data for ART pregnancies was only available as of 2013, analysis of which showed a significant increase in trend over the years with a *p*-value of <0.05.

**Conclusion:**

This study’s findings highlight important public health implications for Malta and the world. The stable rates of preterm and low birth weight, and the decline in stillbirths, suggest improvements in maternal and infant health. However, Malta still lags behind the rest of Europe. This along with the overall increase in the number of births, may be attributed to the growing number of immigrants within the pregnant population, who have specific healthcare needs which need specific attention. These results can help inform public health policies and improve maternity and neonatal services in Malta and regions with similarly increasing immigrant populations.

## Introduction

Perinatal and neonatal health outcomes, including mortality rates, are crucial indicators of a country’s social, health, and economic conditions (1). Many perinatal and neonatal deaths are preventable, making these outcomes key quality indicators for antenatal, obstetric, and postnatal care (2). Monitoring changes in birth rates and adverse outcomes during the perinatal and early childhood periods is essential for improving child health and evaluating interventions at both national and global levels (1, 2).

In 1999, the European Union launched the EURO-PERISTAT network to create a high-quality perinatal health information system for decision-making in European countries. Key indicators such as gestational age, birthweight, neonatal mortality rate, and parity guide healthcare policies. The network has since collected data from 31 European countries, including Malta (2).

**Figure 1:**
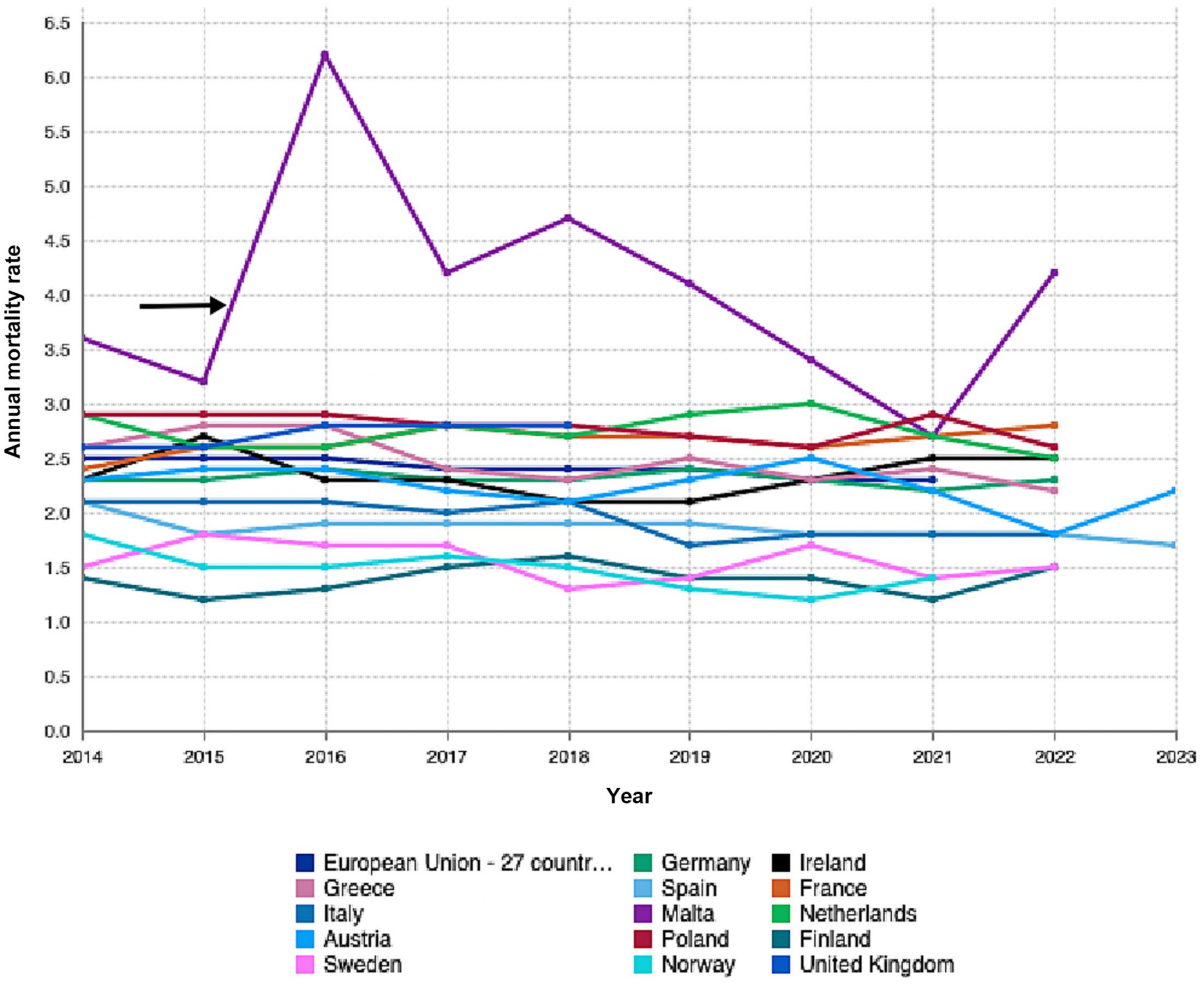
Graph sourced from Eurostat showing annual rates for fetal, peri-and neonatal mortality rates by country of occurrence. Adapted with permission from “Fetal, peri- and neonatal mortality rates by country of occurrence by Eurostat”, by eurostat licensed under CC BY 4.0.

Malta has experienced a steady decline in perinatal and neonatal mortality rates over the past few decades, in line with European trends (2). However, its perinatal mortality rate, around 7.0 per 1,000 total births, is slightly higher than the EU average of approximately 6.0 per 1,000, indicating a need for improvement in maternal and neonatal healthcare outcomes (2) (Figure 1).

This study details perinatal outcomes in Malta from 2008 to 2022, comparing them with rates in other developed countries. The findings aim to provide insights into Malta’s standing among similar European nations, informing future policy and healthcare practices.

The Maltese Islands are an archipelago in the middle of the Mediterranean, with an estimated population of 542,000 as of the year 2022, with children up to the age of five comprising 6.7% of the total population (3). Tertiary neonatal care in Malta is centralized at one general hospital, making it a unique case for studying healthcare systems. Although Malta is part of the EU, it has historically underperformed in health outcomes. Its resource-constrained healthcare system, geographic isolation, and aging population are compounded by a significant influx of immigrants with specific healthcare needs. This context is crucial for developing policies and providing effective perinatal care, especially since immigrants are disproportionately represented in the pregnancy cohort.

This study provides an overview of births and neonatal outcomes in Malta over the past 15 years, examining trends in perinatal and neonatal outcomes. It aims to guide public health policy and resource allocation to reduce mortality rates. Malta’s healthcare system serves as a valuable case study for enhancing maternal and neonatal care in similarly sized European countries.

Studying perinatal outcomes in 5-year epochs over 15 years captures significant trends and provides statistical stability. This approach smooths short-term fluctuations from factors like seasonal variations, facilitating clearer insights into healthcare quality and interventions. It aligns with practices of major health organizations for international comparisons and effectively assesses the long-term effects of health policies, such as improvements in maternal and childcare. This method supports policymakers in evaluating the impact of healthcare reforms over time.

## Methods

### Consent

Data on perinatal outcomes were obtained from the National Obstetric Information System (NOIS) within the Directorate for Health, Information and Research, Malta. This study utilized anonymized, non-identifiable registry data prepared in accordance with General Data Protection Regulation (GDPR) requirements and EUROPERISTAT published data. Ethical review was not required because the research involved secondary use of existing anonymized registry data and did not involve direct interaction with human subjects.

### Population and measurements

Data from NOIS for the 15-year period - 2008 till 2022 were used. NOIS collects information on all in-hospital deliveries occurring in the various hospitals across the Maltese Islands as well as births that incidentally occur outside of the hospital and are then transferred to the hospital. It is estimated that less than 1% of births may be missing from the data registered – these include planned home deliveries that did not require transfer to the hospital for further care.

Data tables for each of the years (2008–2022) included the number of total and live births, gender, numbers of singleton and multiple births, number of live and stillbirths, gestation at birth, birth weight, and number of neonatal deaths. The number of assisted reproduction technology pregnancies, outcomes, and admissions to the neonatal intensive care (NICU) immediately post-delivery were obtained for the years 2018 to 2022 since this data was only available for these years.

### Data analysis

Data were analyzed using SPSS and Microsoft Excel to ensure accurate statistical evaluation of the dataset. The data for each variable were grouped into three 5-year epochs: 2008–2012, 2013–2017, and 2018–2022. This grouping was done to minimize year-on-year fluctuations, which could result from small sample sizes in individual years. By dividing the data into these three epochs, the analysis could provide a more reliable overview of trends over time, ensuring more stable comparisons between groups.

### Handling of variables

Categorical Variables were analyzed using Chi-square tests. The Shapiro Wilk was used to determine whether the distribution of continuous variables satisfied or violated the normality assumption.

Gestational age was analyzed using one-way ANOVA to assess whether the mean gestational age differed significantly across the three time periods.

The difference of two-proportion test was used to compare the proportions of neonates admitted to the NICU in each epoch (i.e., the proportion of NICU admissions in the 2008–2012 period compared to the 2013–2017 and 2018–2022 periods).

### Hypothesis testing

A 0.05 level of significance was used for all statistical tests. If a *p*-value was above 0.05, the null hypothesis was accepted, indicating no significant difference or relationship between the variables and the epochs. On the other hand, a *p* value below 0.05 rejects the null hypothesis.

## Results

### Total births

**Figure 2:**
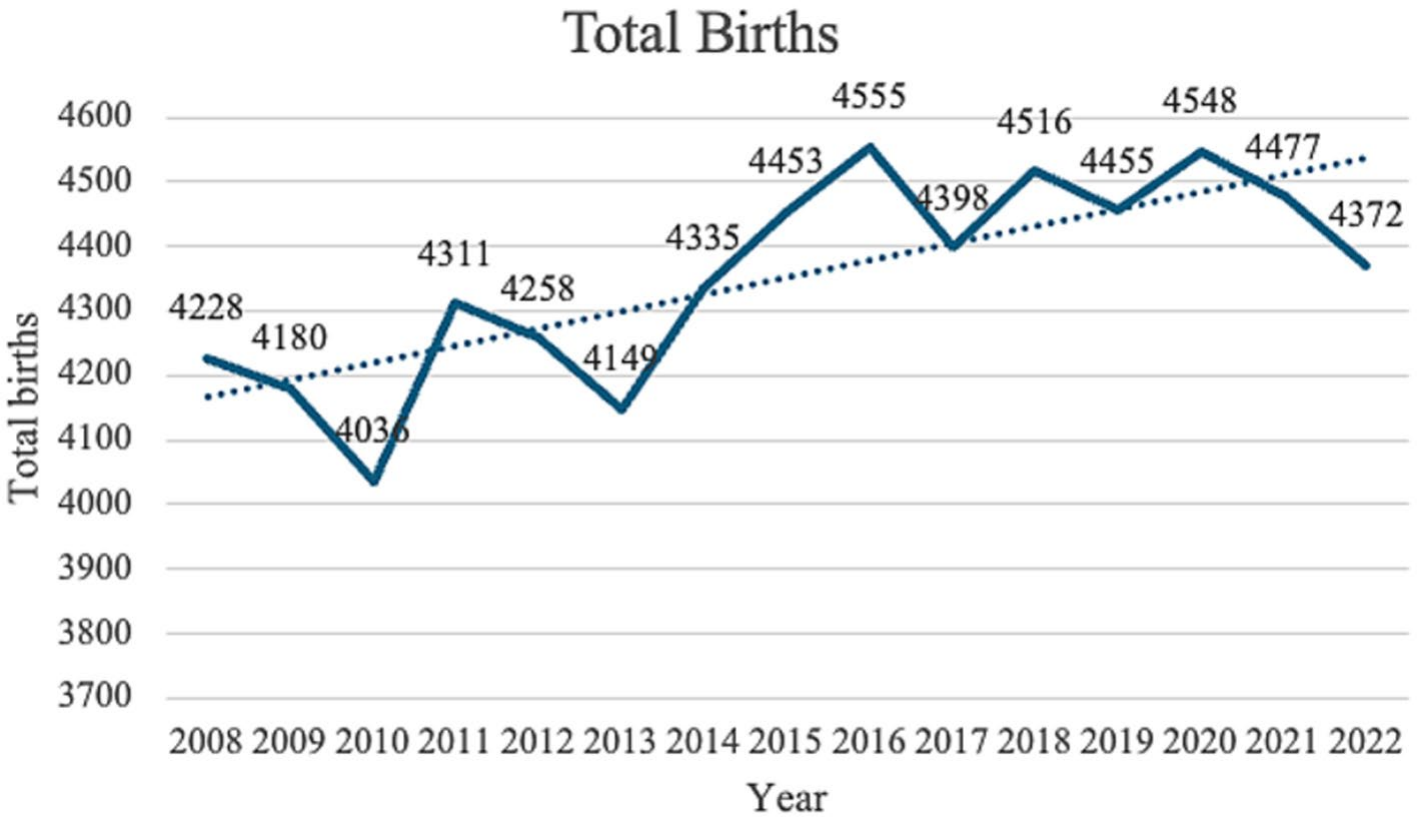
Total births from 2008 till 2022.

When births were categorized within five-year epochs (2008–2012, 2013–2017 and 2018–2022) and the three periods compared, the number of neonates increased significantly throughout the three phases as demonstrated by a *p*-value of 0.008 (Figure 2).

A regression model was fitted to relate the total number of births with time. The regression coefficient (26.5) indicated that on average, the total number births, increases by 26.5 every year. This increment is significant since the *p*-value is 0.001.

### Gender

Comparison of mean frequencies showed that both the number of female and male neonates increased significantly throughout all 5-year phases, with a *p*-value of 0.009 for females and a p-value of 0.022 for males (Table 1). Moreover, throughout the 15 years studied, the proportion of male babies (51.48–52.21%) was invariably significantly larger than the proportion of female newborn babies (47.79–48.22%) for all the individual years with a *p*-value of <0.0001. Male: female ratio throughout the period of study resulted to be 1.08:1.

**Table 1:** Mean frequencies for neonates categorised according to gender.

Variables	Year	Number of deliveries per epoch	Mean	Standard deviation	*p*-value
Female	2008–2012	10,133	2026.60	50.585	**0.009**
	2013–2017	10,462	2092.40	85.967	
	2018–2022	10,853	2170.60	30.320	
Male	2008–2012	10,879	2175.80	63.152	**0.022**
	2013–2017	11,428	2285.60	81.773	
	2018–2022	11,515	2303.00	52.048	

Analysis performed using Chi-square testing. Bold values indicate statistically significant *p*-values.

### Parity

**Figure 3:**
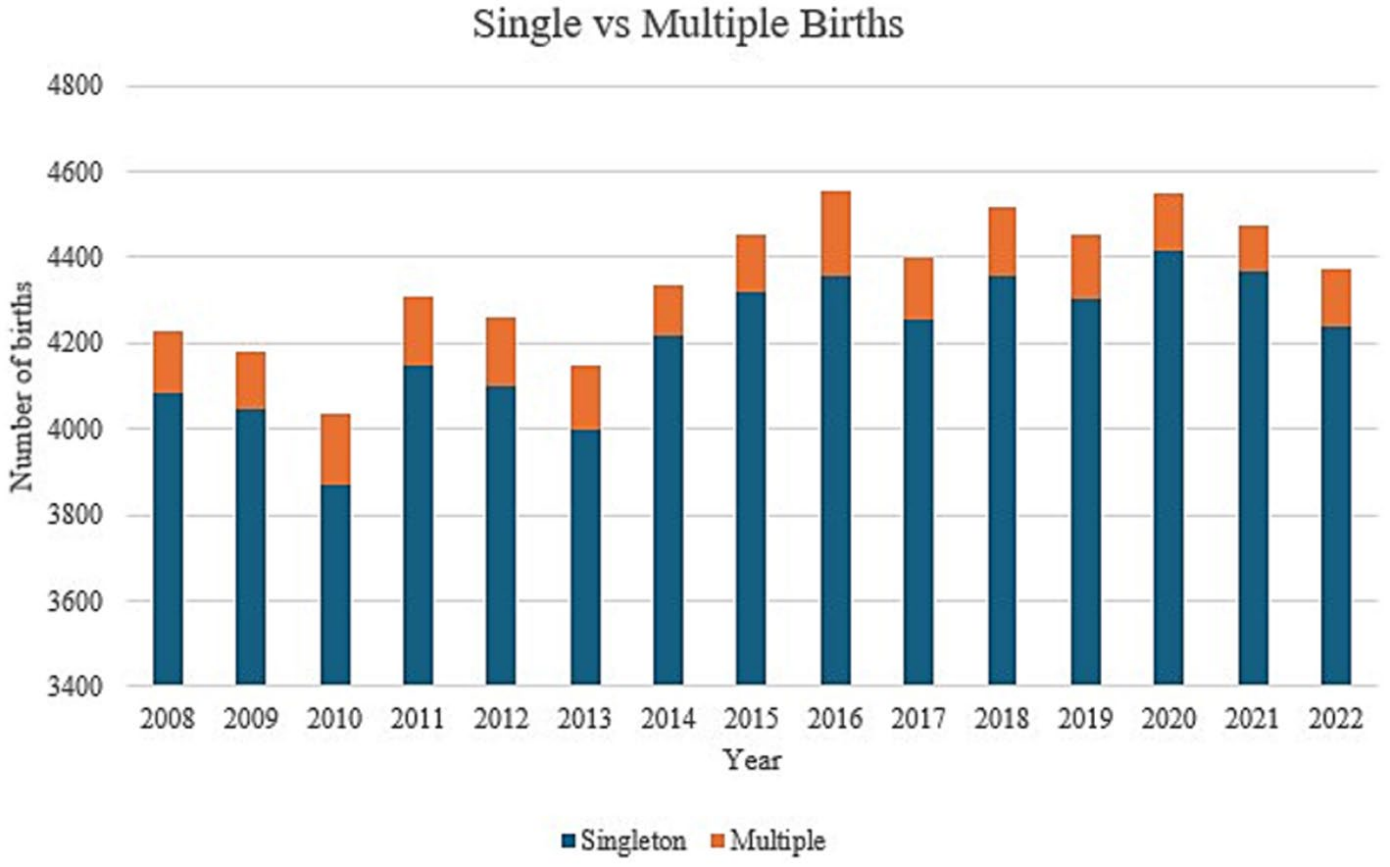
Trends in singleton and multiple births from 2008 till 2022.

**Figure 4:**
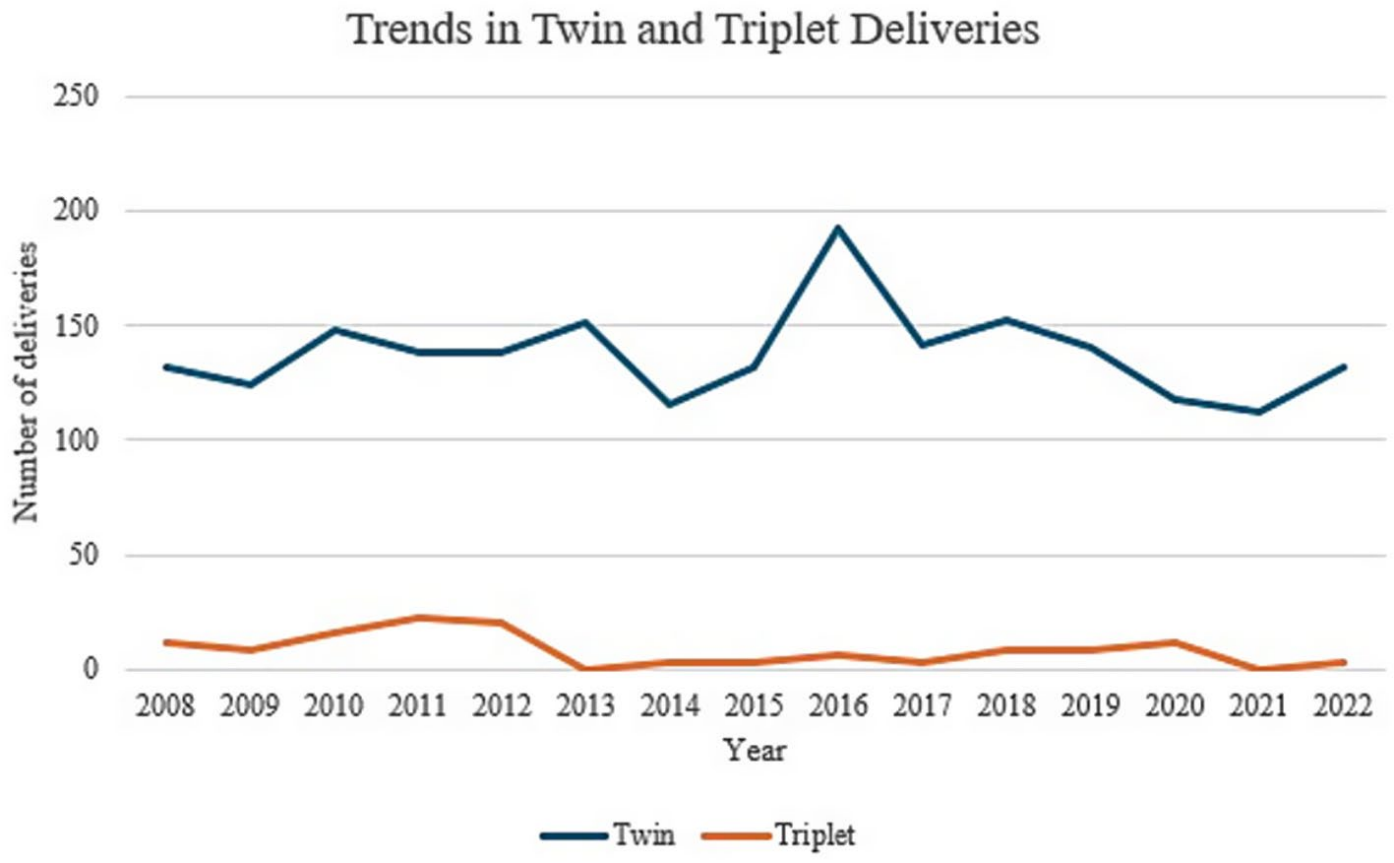
Trends in twin and triplet pregnancies from 2008 till 2022.

**Figure 5:**
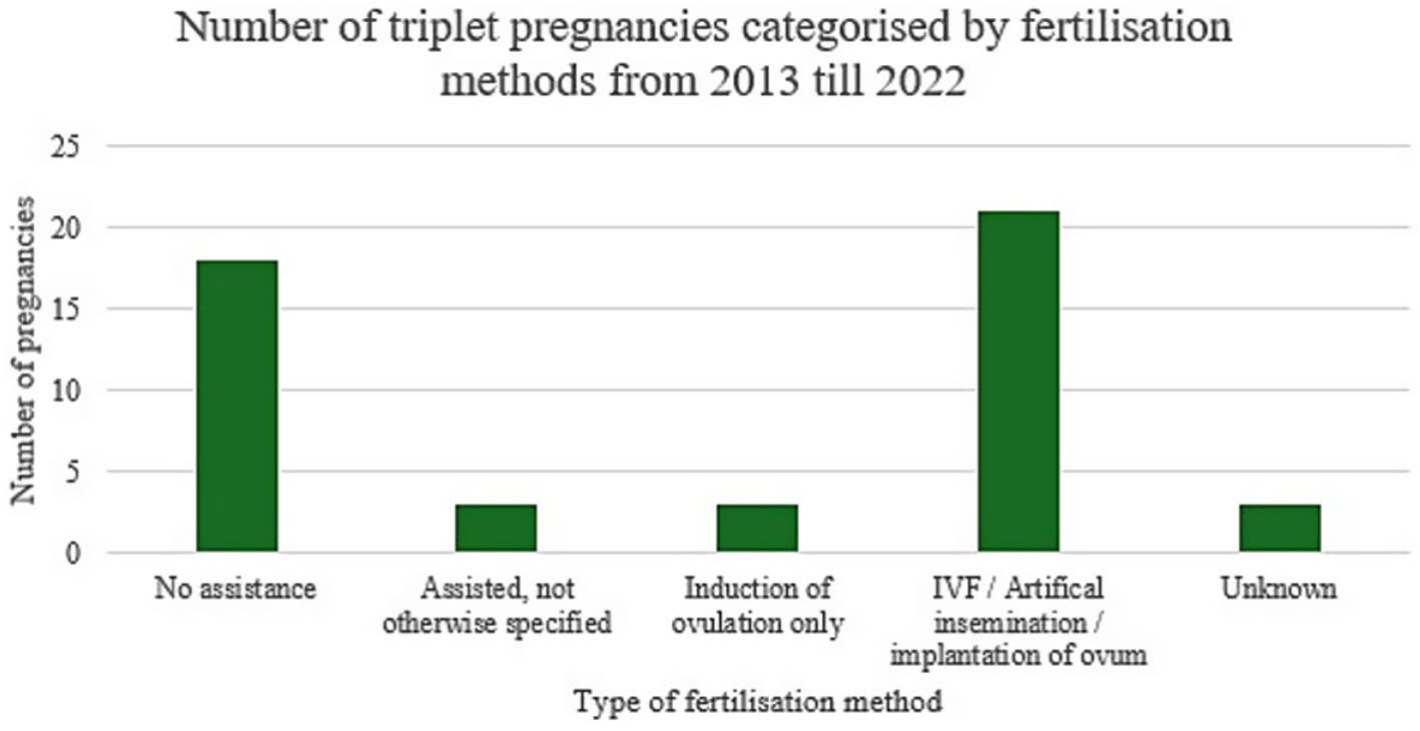
Triplet births categorized by fertilization method.

The number of singleton neonates increased significantly throughout the 3 phases with a p-value of 0.004 (Table 2). On the other hand, no significant difference could be demonstrated for multiple births. Figure 3 demonstrates annual data for singleton and multiple births over the past 15 years. A significant decrease in triplet pregnancies was noted for the 2013–2017 epoch (Table 3, Figure 4). In our population, data for fertilization methods resulting in twin or triplet pregnancies was only available from 2013 onwards. In our population, 56% of triplet births were a result of assisted pregnancies and deliveries compared with 38% occurring spontaneously in non-assisted pregnancies with successful delivery (Figure 5).

**Table 2:** Mean frequencies for neonates categorised according to parity.

Variable	Year	Mean	Standard deviation	*p*-value
Singleton	2008–2012	4050.40	106.359	**0.004**
	2013–2017	4228.40	140.008	
	2018–2022	4336.20	68.240	
Multiple	2008–2012	73.40	5.899	0.813
	2013–2017	74.20	14.618	
	2018–2022	81.00	31.559	

Bold values indicate statistically significant *p*-values.

**Table 3:** Number of twins and triplets for 2008–2022 categorised in five-year time epochs.

Plurality	2008–2012	2013–2017	2018–2022	*p*-value
Twins	680	733	654	0.450
Triplets	81	15	33	**0.002**

Bold values indicate statistically significant *p*-values.

### Assisted reproduction technology (ART) pregnancies and effects on total births

Data for ART pregnancies was only available as of 2013, analysis of which showed a significant increase in trend over the years with a *p*-value of <0.05. The most noteworthy differences were those between the years of 2021 and 2022, with a significant increase for artificial insemination (+2.59% from 2021), IVF and ICSI (+7.12% from 2021) being observed for the year 2022. On the other hand, an 8.75% decrease in ovulation induction was observed in 2022 compared to 2021. There were no significant changes noted for the previous years (Table 4).

**Table 4:** ART pregnancies categorised by type for births from 2018 till 2022.

Type of art	2018 *n* (%)	2019 *n* (%)	2020 *n* (%)	2021 *n* (%)	2022 *n* (%)	Total births
Yes - type not specified	16 (8.79)	18 (12.41)	16 (10.32)	9 (6.82)	11 (5.85)	70
Induction of ovulation	31 (17.03)	33 (22.76)	40 (25.0)	27 (20.45)	22 (11.7)	153
Artificial insemination	8 (4.39)	4 (2.76)	5 (3.23)	5 (3.79)	12 (6.38)	34
IVF, ICSI	127 (69.80)	90 (62.07)	94 (60.64)	91 (68.94)	143 (76.06)	545
Total births	182	145	155	132	188	802

### Live vs stillbirth births

The number of live births was noted to increase significantly throughout the years with a *p*-value of 0.008 (Table 5).

**Table 5:** Changes in stillbirth outcomes from 2008 till 2022.

Outcome	2008	2009	2010	2011	2012	2013	2014	2015	2016	2017	2018	2019	2020	2021	2022
Antepartum stillbirth	20	20	16	23	12	21	25	13	21	18	19	15	22	12	20
Intra partum stillbirth	4	1	1	2	4	1	2	2	1	0	4	0	1	1	1
Stillbirth, not otherwise specified	5	7	1	3	3	0	0	3	1	1	2	1	3	0	0
Total	29	28	18	28	19	22	27	18	23	19	25	16	26	13	21

**Figure 6:**
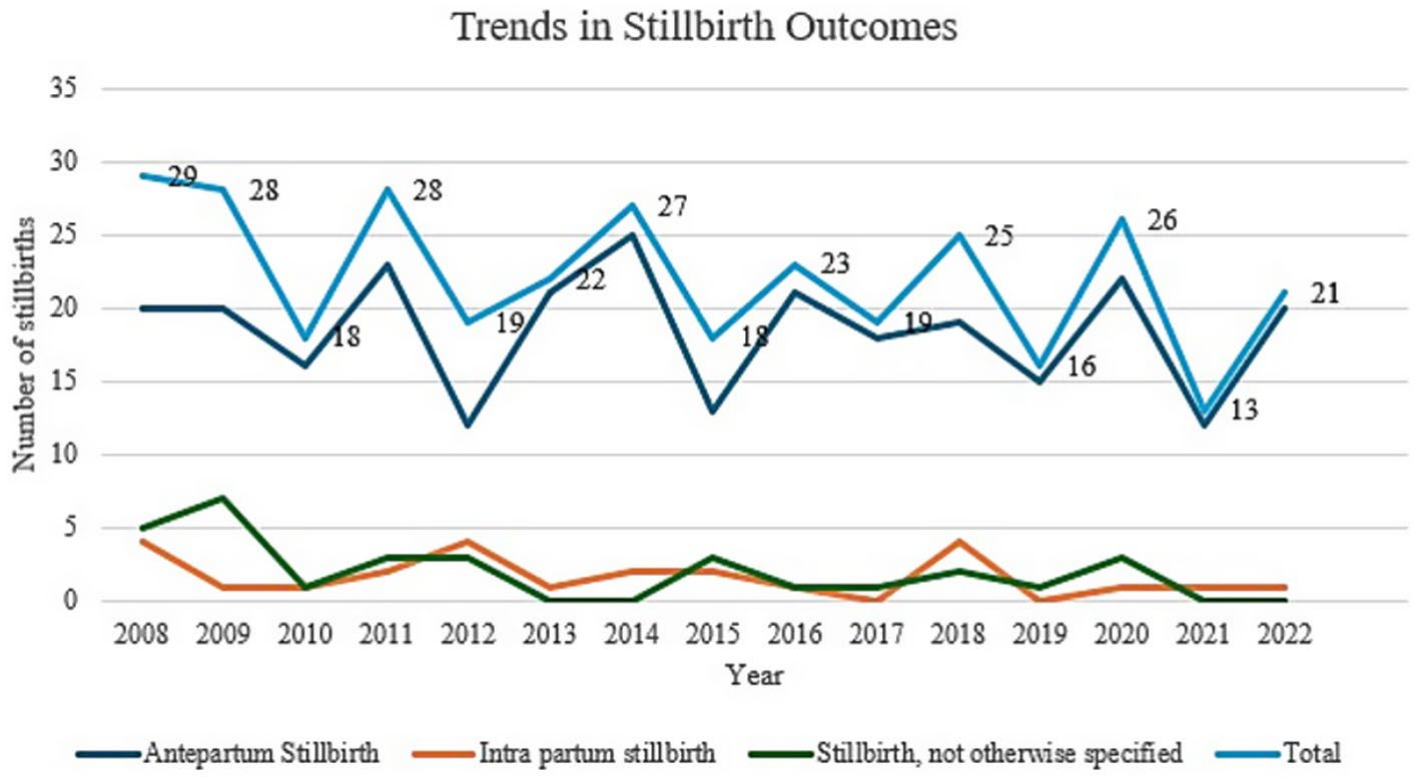
Trends in stillbirth outcomes.

There was no statistically significant decline in stillbirth rate across the years studied. Even though a drop in stillbirth rate was noted between 2018 to 2022, from 7 per 1,000 total births to 5 per 1,000 total births, this was not statistically significant (Table 6). Analysis of trends in stillbirth outcomes (antepartum vs. intrapartum) throughout the years, revealed a significant majority of stillbirths being reported as antepartum as demonstrated in Figure 6.

**Table 6:** Total births categorised into live vs. stillbirth.

Variable	Year	Total births	Mean	Standard deviation	*p*-value	Stillbirth rate (per 1,000 births)
Live	2008–2012	20,891	4178.20	101.812	**0.008**	
	2013–2017	21,781	4356.20	152.062		
	2018–2022	22,249	4449.80	72.234		
Stillbirths	2008–2012	122	24.40	5.413	0.427	5.80
	2013–2017	88	21.80	3.564		4.02
	2018–2022	80	20.20	5.630		3.58

Bold values indicate statistically significant *p*-values.

### Gestation

The absolute number of neonates born between 37 and 40^+6^ weeks was noted to have increased significantly throughout the 15 year period (*p* = 0.003). There were no other significant changes noted for the rest of the gestation categories, with no significant increase in late preterm births across the 3 epochs (*p*-value 0.73).

Despite a decrease in the number of post-term births (beyond 42 weeks’ gestation) over the past 5 years (48 babies 2008–2012, 31 babies 2013–2017, 3 babies 2018–2022), there was no statistical significance between the 3 epochs when comparing term: post-term ratio (*p* = 0.75) and proportion of post-term births out of total births (*p* = 0.087) (Table 7).

**Table 7:** Total births categorised according to gestation.

Variable	Year	Number of babies	Mean	Standard deviation	*p*-value
22–27^+6^ weeks	2008–2012	118	23.60	6.025	0.914
	2013–2017	126	25.20	6.140	
	2018–2022	123	24.60	5.899	
28–31^+6^ weeks	2008–2012	168	33.60	7.021	0.761
	2013–2017	159	31.80	3.834	
	2018–2022	157	31.40	3.130	
32–36^+6^ weeks	2008–2012	1,301	260.20	15.073	0.677
	2013–2017	1,371	274.20	36.561	
	2018–2022	1,330	266.00	16.688	
37–40^+6^ weeks	2008–2012	19,378	3875.60	93.074	**0.003**
	2013–2017	20,203	4040.60	115.589	
	2018–2022	20,751	4150.20	80.014	
41 weeks +	2008–2012	48	9.60	9.915	0.087
	2013–2017	31	6.20	1.924	
	2018–2022	3	0.60	0.894	
Unspecified gestation	2008–2012	0	0.00	0.000	0.148
	2013–2017	0	0.00	0.000	
	2018–2022	3	0.60	0.894	

Bold values indicate statistically significant *p*-values.

### Birthweight

A significant increase in neonates could be observed within the 2000-2999 g category (*p* = 0.027) and the 3,000-3999 g (*p* = 0.007) category over the 15 year period. No significant differences were noted for other birthweight categories throughout the three 5-year phases (Table 8).

**Table 8:** Total births categorised according to birthweight.

Variable	Year	Number of babies	Mean	Standard deviation	*p*-value
BW <500 g	2008–2012	29	5.80	3.347	0.618
	2013–2017	37	7.40	3.715	
	2018–2022	26	5.20	3.701	
BW 500-1499 g	2008–2012	235	47.00	8.515	0.860
	2013–2017	247	49.40	6.914	
	2018–2022	238	47.60	5.727	
1,500-1999 g	2008–2012	283	56.60	9.236	0.727
	2013–2017	276	55.20	15.271	
	2018–2022	305	61.00	10.050	
2000-2999 g	2008–2012	5,519	1103.80	21.076	**0.027**
	2013–2017	5,753	1150.60	62.488	
	2018–2022	5,932	1186.40	29.100	
3,000-3999 g	2008–2012	13,931	2786.20	88.793	**0.007**
	2013–2017	14,579	2915.80	72.040	
	2018–2022	14,859	2971.80	68.379	
4,000-4999 g	2008–2012	971	194.20	10.756	0.458
	2013–2017	1,018	203.60	16.211	
	2018–2022	957	191.40	18.849	
5,000 g+	2008–2012	12	2.40	1.517	0.679
	2013–2017	10	2.00	1.000	
	2018–2022	14	2.80	1.643	
Unspecified birthweight	2008–2012	33	6.60	8.444	0.778
	2013–2017	20	4.00	1.581	
	2018–2022	29	5.80	5.450	

Bold values indicate statistically significant *p*-values.

Most of the 2000-2999 g consisted of newborns born beyond 37 weeks’ gestation, although a significant proportion (14.9%) were newborns between 32 and 36^+6^ weeks’ gestation. For the 3,000-3999 g birthweight category, 98.5% were within the 37^+^ weeks’ category, whilst 1.5% were born between 32- and 36^+6^-weeks’ gestation (Table 9).

**Table 9:** Number of neonates stratified by birthweight and gestation categories.

Birthweight (grams)	22–23^+6^ weeks	24–27^+6^ weeks	28–31^+6^ weeks	32–36^+6^ weeks	37^+^ weeks	Unknown
0–499	51	42	6	0	1	0
500–999	34	183	72	8	3	1
1,000–1,499	0	46	225	139	9	0
1,500–1999	0	3	152	606	103	0
2000–2,499	0	2	14	1,260	1831	0
2,500–2,999	0	0	5	1,306	12,786	0
3,000–3,499	0	1	5	520	27,393	2
3,500–3,999	0	0	1	119	15,279	0
4,000–4,499	0	0	0	26	2,686	0
4,500–4,999	0	0	0	3	231	0
5,000+	0	5	4	9	98	1
Grand total	85	282	484	3,996	60,420	4

### Neonatal deaths

No statistically significant changes were noted for both early and late neonatal deaths throughout the 15 years studied, with a *p*-value of 0.35 (Table 10).

**Table 10:** Number of early and late neonatal deaths from 2008 till 2022.

Variable	Year	Number of live births	*n*	Standard deviation	*p*-value (comparison of means)	Neonatal mortality rate (per 1,000 live births)
Total Number of Neonatal Deaths	2008–2012	20,891	103	2.793	0.350	**4.92**
	2013–2017	21,781	98	4.980		**4.63**
	2018–2022	22,249	85	3.536		**3.92**
Early neonatal	2008–2012	20,891	103	2.702	0.134	
	2013–2017	21,781	98	4.207		
	2018–2022	22,249	85	2.387		
Late neonatal	2008–2012	20,891	16	1.000	0.659	
	2013–2017	21,781	19	1.924		
	2018–2022	22,249	19	1.643		

Bold values indicate statistically significant *p*-values.

**Figure 7:**
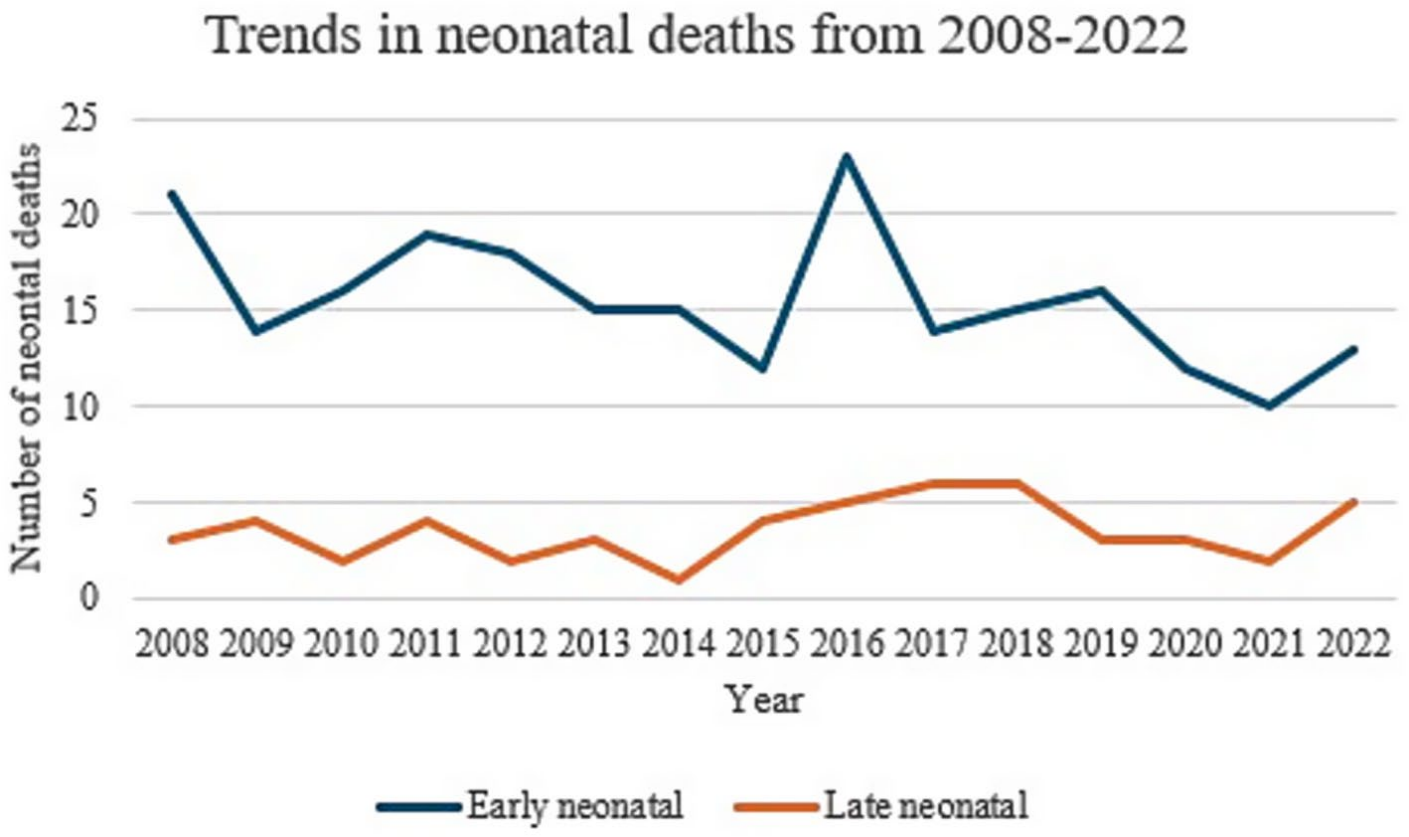
Trends in neonatal deaths from 2008 to 2022.

Therefore, whilst, the neonatal mortality rate appears to have a downward trend, with the lowest being reported for the 2018–2022 epoch at 3.92 per 1,000 live births, compared to 4.92 per 1,000 live births for the years 2008–2012, this was not statistically significant. (Figure 7).

The highest number of early neonatal deaths were reported for the 22–23 weeks category with deaths amounting to 44.7% of the total births within this gestation. On the other hand, the highest number of late neonatal deaths was reported for the 24–27 weeks’ gestation category, at 5.6% of the total births for this category (Table 11).

**Table 11:** All births by gestational age groups and outcome from 2008 till 2022.

GA group	Number of ENDs	% ENDs out of total births per GA group	Number of LNDs	% LNDs out of total births per GA group	Total number of births per GA group
22–23 weeks	38	44.7	3	3.53	85
24–27 weeks	63	22.3	16	5.6	282
28–31 weeks	36	7.4	7	1.4	484
32–36 weeks	40	1	13	0.3	3,996
37 + weeks	55	0.09	13	0.02	60,420
Unknown gestation	1	25	1	25	4
Total	233	0.36	53	0.08	65,271

GA, gestational age; END, early neonatal deaths; LND, late neonatal deaths.

### Trends in admission to the neonatal intensive care unit immediately after delivery

Data for this variable was only available from 2018 onwards. The rate of admissions varied from 6.88 to 9.06% of the total live births. Comparing the year 2019 (the highest proportion of admissions) with 2021 (the lowest proportion of admissions), the resulting difference in admissions is significant (*z* score 3.8, *p*-value of 0.0001) (Table 12).

**Table 12:** Number of admissions to NICU since 2018 expressed as a percentage out of total live births.

Year	Live births	Admitted to NICU	NICU admissions *Z*-score	% of total live births admitted
2018	4,491	360	0.36	8.02
2019	4,439	402	1.53	9.06
2020	4,522	335	−0.34	7.41
2021	4,464	307	−1.12	6.88
2022	4,333	332	−0.42	7.66

## Discussion

Neonatal and childhood mortality remains a significant global challenge up to this day, with an estimated death of 2.4 million newborns within their first month of life reported by UNICEF in 2023 alone, most of which were deemed preventable. This represents a significant loss of life and is a huge burden on communities, especially in the context of developing countries in which most of these deaths have been observed to occur (4, 5).

Testament to ongoing commitments of organizations, governments and healthcare professionals, the United Nations Inter-Agency Group for Child Mortality Estimation has reported in 2023 a global decline in the under-5-mortality rate by 51% since the year 2000 (6). Despite this, huge disparities in neonatal mortality still exist, ranging from <5 deaths per 1,000 live births in developed countries, to >35 deaths per 1,000 live births in low-income and developing countries. This emphasises the importance of having accessible high-quality healthcare, in both antenatal and postnatal settings; including the availability of services to care for the preterm and sickest newborns, to curtail preventable child deaths in every community (6–8).

Analysis of the total number of births over the past 15 years, revealed a significant increase in birth rate. Malta has experienced unprecedented changes in population size and structure, with an estimated 21% increase in resident population between 1998 and 2017, and a total foreign population of 115,449 inhabitants as of 2021, with foreigners between the years of 30 and 39 years of age comprising the biggest share (3, 9). Therefore, despite a decrease in deliveries to Maltese women, the resulting net immigration of foreigners could have been a significant factor in contributing toward an increase in the overall birth rate (9, 10). Within the European Union region, crude birth has been shown to overall decrease since 2021 to 9.1 live births per 1,000 persons (in contrast to 10.6 live births per 1,000 persons in 2008), although variations in this pattern were noted across countries, with sixteen country states reporting a decreasing trend and ten states reporting increasing trends. Malta fared the lowest total fertility rate as of 2022 at 1.15 live births per woman, in contrast to France with a reported 1.79 live births per woman. Moreover, 22% of children in the EU were reported to be born to foreign-born mothers. Malta was reported to have the highest increase in live births from foreign-born mothers at 33% (previously at 11% in 2013) from across EU member states, followed by Portugal (8 percentage points from 24 to 16%), and Spain, Cyprus and Slovenia for which increases of 7 percentage points were recorded; thus, explaining increasing trends in births for the Maltese population (11, 12). Analysis of gender revealed a stable male-to-female ratio of 1.08:1 throughout the period studied. Male: female ratio in the EU was reported to be at 1.055 in 2021 (13).

Multiple pregnancies pose a high risk of morbidity and mortality for both mothers and their newborns, requiring timely obstetric decisions and a balance of maternal and neonatal complications (14, 15). In our study, whilst the number of singleton neonates was noted to increase significantly throughout the 3 phases, no significant difference could be demonstrated for multiple births. Spontaneous triplet pregnancies are known to be relatively rare events occurring in 1/8000 pregnancies, though an upsurge was recorded worldwide secondary to the use of assisted reproductive techniques between the 1980s and 1990s. Its incidence was noted to thereafter decline as recommendations to minimize multiple embryo transfers were made (16, 17). These changes in trends have also been mirrored locally, as demonstrated in our study, as the implementation of the Embryo Protection Act came into force in 2012 with a legislative amendment regarding embryo freezing in 2018, with more couples having one embryo transferred rather than multiple. In our population, 56% of triplet births were a result of assisted pregnancies and deliveries compared with 38% occurring spontaneously in non-assisted pregnancies with successful delivery (18, 19).

A significant increase in absolute number of term births (37^+^ weeks) and in birthweight (2000-3999 g) was noted for the Malta population over the past 15 years, with no significant change in preterm and low birthweight births, reflecting preterm birth rates over the last decade at a global level. Interestingly, no increase in late preterm birth rates has been observed for Malta, as opposed to changes worldwide (20). Despite no statistical significance noted in the number of post-term babies over the years, a notable decrease in the number of babies being born beyond 42 weeks of gestation was observed for the past 5 years, which might suggest an increase in the number of labor induction practices. This reflects the increasing practice of labor induction over the past several decades in developed countries secondary to recommendations by various professional bodies outlining risks of spontaneous post-term labor vs. induction of labor (21–23). In developed countries, the proportion of infants delivered by induction is thought to be as high as 1 in 4 deliveries, quoted at up to 33% of total deliveries in some countries with considerable intercountry variations. Induction rates in Malta were quoted to be as high as 28.9% in 2015 (21–23).

Despite an overall worldwide decline in infant mortality rates of up to 52% over the past 22 years, the percentage of neonatal deaths has been reported to increase over time from 41% in the year 2000 to 47% in 2022, despite a decrease in the absolute number of neonatal deaths by 44% since 2000 (6). In our study, no significant changes in trends were noted for both early and late neonatal deaths throughout the 15 years. This stable trend in death rate can be attributed to various reasons including population change over the past few years, with high immigration patterns from conflict-zone countries (24). Various research has demonstrated immigration as an important determinant of health, with female immigrants being considered a vulnerable population, with limited healthcare access resulting in worse neonatal and perinatal outcomes compared to those of native-origin women in host countries (24–27). Differences in termination of pregnancy legislation between countries, especially within the context of pregnancies with congenital anomalies, might also play a role in affecting neonatal death rate. This argument is supported by a study published in 2023 by Tierny et al. (29), evaluating the effects of changes in abortion legislation in 2018 on admissions to paediatric intensive care in Ireland after its introduction, which demonstrated a modest reduction in live births with significant congenital anomalies since the introduction of the 2018 Termination of Pregnancy Act as represented by the numbers of these infants presenting to paediatric intensive care unit. On the other hand, no statistically significant decline in perinatal and neonatal mortality rates was reported. Tierny et al. (29), go on to argue that major congenital anomalies remain the major cause of both early and late neonatal deaths, with a significantly reduced perinatal mortality rate when this is corrected for major congenital anomalies. This is interesting and warrants deeper investigation given that like Ireland, Malta has restrictive laws when it comes to non-medical termination of pregnancy (28, 29).

In our study, stillbirth rates showed a downward trend from 7 per 1,000 total births in 2008 to 5 per 1,000 total births in 2022. Despite this downward trend, statistical significance was not reached and the stillbirth rate reported in our study is still high when compared to other countries, with a reported stillbirth rate below 2.5 stillbirths per 1,000 total births for most European countries, and 4 stillbirths per 1,000 total births in England and Wales. Data on stillbirths and socioeconomic status from routine systems showed widespread and consistent socioeconomic inequalities in stillbirth rates in Europe, but further research is needed to better understand differences between countries in the magnitude of the socioeconomic gradient (30–33).

The rate of admissions to NICU from 2018 to 2022 for our population ranged from 6.88% in 2021 (68 per 1,000 live births) to 9.06% of the total live births in 2019 (91 per 1,000 live births). Comparing the year 2019 (the highest proportion of admissions) with 2021 (the lowest proportion of admissions), the resulting difference in admissions is significant (z score 3.8, *p*-value of 0.0001). This was an interesting finding as overall NICU admissions seemed to remain stable, although no information was available as of pre-2018 to comment on whether assisted reproduction technologies have led to an increase in admission rates. Still, admission rates for term babies have been quoted to be less than 5.2% of total births in the year 2016 (34). The stable admission rates despite the COVID-19 pandemic in 2020 were an interesting finding, given that other countries and studies reported higher NICU admission numbers during the pandemic secondary to its profound negative maternal and neonatal impacts secondary to lockdown measures, maternal health complications and disruptions in prenatal care (35, 36). This observation in admission rates could possibly be a reflection of the recent modifications and implementation of stringent admission criteria during the period studied, such as increasing the cord pH thresholds for admission. Moreover, there has also been an improvement in obstetric diagnosis with some cases being transferred to tertiary centres abroad antenatally for delivery overseas.

## Limitations

Data supplied for birthweight and gestation were already categorized independently of each other, thus it was not possible to categorize individual infants into small, appropriate, and large gestational age categories. Some data for birthweight (0.006%) and gestation (0.005%) was missing. Information on admissions to NICU and ART was only available as of 2018.

The data set lacked information maternal on country of origin and maternal education, which prevented us from analysing the implications of these factors on health outcomes. Factors such as socio-economic status, healthcare access, cultural practices, and language barriers can significantly impact these outcomes. Future research should prioritize comprehensive data collection from these particular groups.

The absence of long-term data on Assisted Reproductive Technology (ART) outcomes is a limitation of this study. The collection of ART-specific data and extended follow-up periods is vital for informing healthcare provision and best practice.

## Conclusion

This study reports perinatal outcomes in Malta from 2008 to 2022, divided into three five-year epochs (2008–2012, 2013–2017, 2018–2022). The rate of stillbirth, preterm births and low birthweight deliveries did not show a statistically significant difference between these epochs. There has been an increase of 26.5 births per year (p 0.001) throughout the period studied. The underlying factors that have led to this increase, need further study. This increasing trend has important implications for access to care and for understanding the specific needs of pregnant women and their offspring.

Additionally, the use of Assisted Reproductive Technologies (ART) is increasing in Malta and other parts of the world, impacting perinatal care. This also requires specialized care for high-risk pregnancies. These developments are particularly relevant to policymakers and healthcare providers. Therefore, it is essential to thoroughly understand these implications when designing perinatal healthcare services and when organising data collection and monitoring systems.

## Data Availability

The original contributions presented in the study are included in the article/supplementary material, further inquiries can be directed to the corresponding author/s.

## References

[1] Zeitlin J Wildman K Bréart G Alexander S Barros H Blondel B. PERISTAT: indicators for monitoring and evaluating perinatal health in Europe. Eur J Pub Health. (2003) 13:29–37. doi: 10.1093/eurpub/13.suppl_1.29, PMID: 14533746

[2] European perinatal health report. (2010) [Internet]. Euro Peristat. (2013). Available online at: https://www.europeristat.com/publications/european-perinatal-health-report-2010/

[3] Malta National Statistics Office. Regional statistics malta 2023 Edition. (2023). Available online at: https://nso.gov.mt/wp-content/uploads/Regional-Statistics-Malta-2023-Edition.pdf

[4] United nations inter-Agency Group for Child Mortality Estimation (UN IGME). Levels and trends in child mortality: Report. New York: (2020).

[5] United Nations Inter-Agency Group for Child Mortality Estimation (UN IGME). A neglected tragedy: the global burden of stillbirths. Unicef.org Available online at: https://www.unicef.org/reports/neglected-tragedy-global-burden-of-stillbirths-2020 (2020).

[6] Estimates developed by the UN Inter-agency Group for Child Mortality Estimation. (2023). Available online at: https://childmortality.org/wp-content/uploads/2024/03/UNIGME-2023-Child-Mortality-Report.pdf

[7] Arora A. Levels and trends in child mortality, Unicef.org Available online at: https://data.unicef.org/resources/levels-and-trends-in-child-mortality-2024/ (2024)

[8] UN Children’s FUND, WHO, World Bank Group, UN Department of Economic and Social Affairs Population Division, UN Economic Commission for Latin America and the Caribbean Population Division. (2021). Available online at: https://www.who.int/publications/m/item/levels-and-trends-in-child-mortality-report-2021

[9] Prevalence charts and tables Europa. eu. Available online at: https://eu-rd-platform.jrc.ec.europa.eu/eurocat/eurocat-data/prevalence_en (2018)

[10] England K Buttigieg S. The impact of demographic changes in Malta on health and the health system over the past two decades. Centres for Disease Control and Prevention. (2019) Available online at: https://www.um.edu.mt/library/oar/handle/123456789/49262

[11] Demography of Europe – 2023 edition - Interactive publications - Eurostat [Internet]. Europa.eu. Available online at: https://ec.europa.eu/eurostat/web/interactive-publications/demography-2023

[12] Statistics explained [Internet]. Europa.eu. (2023). Available online at: https://ec.europa.eu/eurostat/statistics-explained/index.php?title=Fertility_statistics

[13] TRADING ECONOMICS. European Union - Sex ratio at birth (male births per female births). 20250427T06: 36: 00.00Z. (2023). Available online at: https://tradingeconomics.com/european-union/sex-ratio-at-birth-male-births-per-female-births-wb-data.html

[14] Bernal Claverol M Aracil Moreno I Ruiz Minaya M Fernández Muñoz M Reyes Angullo ZR González Navarro P. Maternal, perinatal and neonatal outcomes of triplet pregnancies according to chorionicity: a systematic review of the literature and meta-analysis. J Clin Med. (2022) 11:1871. doi: 10.3390/jcm13061793 35407479 PMC8999732

[15] Hayata E Nakata M Morita M. Time trend analysis of perinatal mortality, stillbirth, and early neonatal mortality of multiple pregnancies for each gestational week from the year 2000 to 2019: A population-based study in Japan (2022) 17. doi: 10.1371/journal.pone.0272075 PMC9312402 35877663

[16] Martin JA Osterman MJ Thoma ME. Declines in triplet and higher-order multiple births in the United States, 1998–2014. NCHS Data Brief. (2016) 243:1–8. Available online at: https://www.cdc.gov/nchs/products/databriefs/db243.htm 27139731

[17] Benirschke K Kim CK. Multiple pregnancy. N Engl J Med. (1973) 288:1329–36. doi: 10.1056/NEJM197306212882505, PMID: 4267537

[18] Vella S. Widened access and scope of in vitro fertilisation laws, amid controversy European Social Policy Network (2022) ESPN Flash Report 2022/44. European Commission.

[19] Grech V Gatt M. Assisted reproductive technology and multiple pregnancies in Malta — a population-based study. Early Hum Dev. (2021) 157:105378. doi: 10.1016/j.earlhumdev.2021.105378 33906003

[20] Karnati S Kollikonda S Abu-Shaweesh J. Late preterm infants - changing trends and continuing challenges. Int J Pediatr Adolesc Med. (2020) 7:36–44. doi: 10.1016/j.ijpam.2020.02.006, PMID: 32373701 PMC7193066

[21] Caruana M Giacchino T Borg M Baron YOM. Induction of labour at mater Dei hospital Malta. Eur J Obstetrics Gynaecol Reproductive Biol. (2016) 206:e23. doi: 10.1016/j.ejogrb.2016.07.087

[22] Caughey AB Sundaram V Kaimal AJ Cheng YW Gienger A Little SE. Maternal and neonatal outcomes of elective induction of labour. Evidence Report/Technology Assessment No. 176. Agency for Healthcare Research and Quality (2009). Available online at: https://pubmed.ncbi.nlm.nih.gov/19408970/PMC4781541 19408970

[23] Seijmonsbergen-Schermers AE van den Akker T Rydahl E Beeckman K Bogaerts A Binfa L. Variations in the use of childbirth interventions in 13 high-income countries: a multinational cross-sectional study. PLoS Med. (2020) 17:e1003103. doi: 10.1371/journal.pmed.1003103, PMID: 32442207 PMC7244098

[24] Behboudi-Gandevani S Bidhendi-Yarandi R Panahi MH Mardani A Prinds C Vaismoradi M. Perinatal and neonatal outcomes in immigrants from conflict-zone countries: a systematic review and Meta-analysis of observational studies. Front Public Health. (2022) 10, 766943. doi: 10.3389/fpubh.2022.766943, PMID: 35359776 PMC8962623

[25] Hili C Savona-Ventura C Xuereb RB. The perinatal outcomes of Maltese and sub-Saharan African migrant women: a comparative. Retrospective Study Creative Nursing. (2023) 13:IJC-2022-0012.R1. doi: 10.1891/IJC-2022-0012

[26] Savona-Ventura C Buttigieg GG Gatt M. Obstetric outcomes in immigrants of African nationality. Int J Risk Safety Med. (2009) 21:147–52. doi: 10.3233/JRS-2009-0473

[27] World Health Organization (WHO). Trends in maternal mortality 2000–2017. Geneva: World Health Organization (2019).

[28] San Lazaro Campillo I Manning E Corcoran P Keane J McKernan J Escanuela Sanchez T. On behalf of the Perinatal Mortality National Clinical Audit Governance Committee. Perinatal Mortality National Clinical Audit in Ireland Annual Report 2021. Cork: National Perinatal Epidemiology Centre (2023).

[29] Tierney N Healy M Lyons B. Changes in abortion legislation and admissions to paediatric intensive care in Ireland. Clin Ethics. (2024) 19:47–53. doi: 10.1177/14777509231196700

[30] WHO. Indicator Metadata Registry Details. (2023). Available online at: https://www.who.int/data/gho/indicator-metadata-registry/imr-details/2444#:~:text=Definition%3A

[31] UNICEF. Stillbirths and stillbirth rates. UNICEF DATA. (2023). Available online at: https://data.unicef.org/topic/child-survival/stillbirths/ (Accessed on August 8, 2024).

[32] Stillbirth statistics. [Internet]. Tommys.org. [cited 2024 Aug 8]. Available online at: https://www.tommys.org/baby-loss-support/stillbirth-information-and-support/stillbirth-statistics. (2022).

[33] Zeitlin J Mortensen L Prunet C Macfarlane A Hindori-Mohangoo AD Gissler M. Socioeconomic inequalities in stillbirth rates in Europe: measuring the gap using routine data from the euro-Peristat project. BMC Pregnancy Childbirth. (2016) 16:15. doi: 10.1186/s12884-016-0804-4, PMID: 26809989 PMC4727282

[34] Borg R Dimech MA Xuereb S Muscat Baron Y. Term admissions to neonatal intensive care a Maltese observational study. Malta Medical School Gazette. (2018) 2, 4–9. Available online at: https://www.um.edu.mt/library/oar/handle/123456789/37415

[35] Harrison W Goodman D. Epidemiologic trends in neonatal intensive care, 2007-2012. JAMA Pediatr. (2015) 169:855–62. doi: 10.1001/jamapediatrics.2015.1305, PMID: 26214387

[36] Jegatheesan P Narasimhan SR Huang A Nudelman M Song D. Higher NICU admissions in infants born at ≥35 weeks gestational age during the COVID-19 pandemic. Front Paediatrics. (2023) 11:1206036. doi: 10.3389/fped.2023.1206036, PMID: 37484778 PMC10360125

